# Vasculoprotective properties of plasma lipoproteins from brown bears (*Ursus arctos*)

**DOI:** 10.1016/j.jlr.2021.100065

**Published:** 2021-03-11

**Authors:** Matteo Pedrelli, Paolo Parini, Jonas Kindberg, Jon M. Arnemo, Ingemar Bjorkhem, Ulrika Aasa, Maria Westerståhl, Anna Walentinsson, Chiara Pavanello, Marta Turri, Laura Calabresi, Katariina Öörni, Gérman Camejo, Ole Fröbert, Eva Hurt-Camejo

**Affiliations:** 1 Division of Clinical Chemistry, Department of Laboratory Medicine, Karolinska Institutet, Stockholm, Sweden; 2 Translational Science & Experimental Medicine, Research and Early Development, Cardiovascular, Renal and Metabolism (CVRM), BioPharmaceuticals R&D, AstraZeneca, Gothenburg, Sweden; 3 Metabolism Unit, Department of Medicine, Karolinska Institutet, Stockholm, Sweden; 4 Theme Inflammation and Infection, Karolinska university Hospital, Stockholm, Sweden; 5 Norwegian Institute for Nature Research, Trondheim, Norway; 6 Swedish University of Agricultural Sciences, Department of Wildlife, Fish, and Environmental Studies, Umeå, Sweden; 7 Department of Forestry and Wildlife Management, Inland Norway University of Applied Sciences, Campus Evenstad, Koppang, Norway; 8 Department of Community Medicine and Rehabilitation, Umeå University, Umeå, Sweden; 9 Division of Clinical Physiology, Department of Laboratory Medicine, Karolinska Institutet, Stockholm, Sweden; 10 Centro Enrica Grossi Paoletti, Dipartimento di Scienze Farmacologiche e Biomolecolari, Università degli Studi di Milano, Milan, Italy; 11 Atherosclerosis Research Laboratory, Wihuri Research Institute, Helsinki, Finland; 12 Örebro University, Faculty of Health, Department of Cardiology, Örebro, Sweden

**Keywords:** *Ursus arctos*, hibernation, lipids, apoB, lipoproteins, proteoglycans, cholesterol efflux, atherosclerosis, triglycerides, LDL, AD, aqueous diffusion, apoAI, apolipoprotein AI, apoB, apolipoprotein (B), apoE, apolipoprotein E, CE, cholesteryl ester, CEC, cholesterol efflux capacity, CETP, cholesteryl ester transfer protein, CM, chylomicron, PG, proteoglycan, PL, phospholipid, SR-BI, scavenger receptor class B type I, TC, total cholesterol, TG, triglyceride, UC, unesterified cholesterol

## Abstract

Plasma cholesterol and triglyceride (TG) levels are twice as high in hibernating brown bears (*Ursus arctos*) than healthy humans. Yet, bears display no signs of early stage atherosclerosis development when adult. To explore this apparent paradox, we analyzed plasma lipoproteins from the same 10 bears in winter (hibernation) and summer using size exclusion chromatography, ultracentrifugation, and electrophoresis. LDL binding to arterial proteoglycans (PGs) and plasma cholesterol efflux capacity (CEC) were also evaluated. The data collected and analyzed from bears were also compared with those from healthy humans. In bears, the cholesterol ester, unesterified cholesterol, TG, and phospholipid contents of VLDL and LDL were higher in winter than in summer. The percentage lipid composition of LDL differed between bears and humans but did not change seasonally in bears. Bear LDL was larger, richer in TGs, showed prebeta electrophoretic mobility, and had 5–10 times lower binding to arterial PGs than human LDL. Finally, plasma CEC was higher in bears than in humans, especially the HDL fraction when mediated by ABCA1. These results suggest that in brown bears the absence of early atherogenesis is likely associated with a lower affinity of LDL for arterial PGs and an elevated CEC of bear plasma.

The plasma lipoproteins of many mammalian species can be separated into corresponding classes of chylomicrons (CMs), VLDL, LDL, and HDL (1). The lipoproteins in each class share similar density ranges but have species-specific lipid and apolipoprotein composition. Furthermore, the association between plasma lipid lipoprotein levels and the susceptibility to atherosclerosis development vary within a species (1). Several species of the genus *Ursus*, which hibernate during winter, have levels of plasma total cholesterol (TC), triglycerides (TGs), and phospholipids (PLs) that are much higher than those found in healthy humans (2, 3, 4, 5). Interestingly, European brown bears (*Ursus arctos*), which have high TC, LDL-C, and TG, apparently do not develop atherosclerosis (6). In bears, plasma lipids circulate as lipoproteins with densities similar to those of human VLDL, LDL, and HDL (2, 3, 4, 5). Paradoxically, the plasma levels of lipids and lipoproteins in brown bears are higher in hibernation (fasting) than when these animals are active and have access to food (2, 3, 4, 6). The field and laboratory activities of the Scandinavian Brown Bear Research Project (https://bearproject.info) have provided access to blood and tissue samples taken from the same animals during hibernation in winter (February) and when active in summer (June). Previous studies from this project documented the pronounced metabolic differences between winter and summer and provided valuable translational information for the relation of these changes with human disease (2). Most evident are differences in metabolic parameters related to energy balance, hematological adaptation, endocrine status, kidney function, protein synthesis, protein degradation, and plasma lipids (2, 5, 6). High plasma levels of TC, TG, LDL-C, HDL-C, and circulating lipases are associated with hibernation in both black and brown bears (3, 4, 6). Interestingly, histopathological examination of the descending coronary artery and aortic arches of 12 adult free-ranging bears revealed no sign of foam cell infiltration, fatty streaks, or late lesions. Arterial specimens from bears had similar morphology to muscular arteries as found in nonatherosclerotic healthy humans (6).

The lack of association between high plasma lipid levels and early stage atherosclerosis development in free-ranging brown bears is poorly understood, despite its translational potential to the human conditions. In the present study, we examined the lipoprotein composition and functions from the same free-ranging Swedish brown bears during hibernation and in their active state and compared them with those of apparently healthy humans. Apolipoprotein B (apoB)-containing lipoproteins (VLDL, remnants, LDL, and lipoprotein (a)), which enter the arterial intima can become bound to proteoglycans (PGs) in the extracellular matrix. This process initiates plaque formation as described in the “*response to retention*” hypothesis of early atherogenesis (7, 8). Conversely, apolipoprotein AI (apoAI)-containing lipoprotein (HDL) seem to have antiatherogenic properties by promoting cholesterol efflux from peripheral cells [cholesterol efflux capacity (CEC)] (9).

In the present study, we report on the lipoprotein composition and two functional properties that appear to modulate early atherogenesis in humans and preclinical mammalian models: the association of LDL with arterial PGs and the capacity of plasma to remove cholesterol from extrahepatic cells (CEC).

## Materials and methods

### Animals

Bear capture, anesthesia, and sampling procedures have been described previously (5, 6, 10). In summary, to trace their movement, the bears were equipped with collars with a Global Positioning System collars and very high frequency transmitter implants. A team from the Scandinavia Brown Bear Research Project collected samples from the same subadult Swedish brown bears twice a year, during hibernation (February 2011 and 2012) and the active state (June 2011 and 2012). All capture and handling protocols were approved by the Swedish Ethical Committee on Animal Research (application numbers C212/9 and C47/9) and the Swedish Environmental Protection Agency. All captures were carried out in Dalarna and Gävleborg counties, south-central Sweden. Blood was drawn from the anesthetized animal and collected in tubes containing EDTA as anticoagulant. The coded blood samples were kept in refrigeration (1–2 h) during rapid transport (helicopter) to the field laboratory, where plasma was prepared by centrifugation (2,000 *g*, 4°C, 10 min). EDTA plasma samples were frozen and shipped on dry ice to our laboratory, where they were stored at −80°C.

### Materials and reagents

RPMI medium, high glucose DMEM, MEM, trypsin-EDTA, gentamicin, and penicillin-streptomycin were purchased from Thermo Fisher Scientific Europe BV, The Netherlands (Stockholm, Sweden). Tissue culture flasks, plates, scrapers, and tubes were purchased from Thermo Fisher Scientific Europe BV, The Netherlands (Stockholm, Sweden) or Falcon (Lincoln, NY). Polyethylene glycol solution, FBS, ACAT inhibitor Sandoz 58-035, DMSO, BSA, and 8-(4-chlorophenylthio)-cAMP salt were purchased from Sigma-Aldrich® (Stockholm, Sweden). [1,2-^3^H(N)]-cholesterol and Ultima Gold™ were purchased from PerkinElmer® (Upplands Väsby, Sweden). Block lipid transporter 1 was purchased from ChemBridge Corporation (San Diego, CA). Human recombinant apoAI was purchased from tebu-bio (Le Perray-en-Yvelines, France).

Serum samples (n = 14) were randomly selected from a large cohort of healthy subjects enrolled in the Swedish physical activity and fitness cohort study ((11); ethical permission: EPN Umeå, Dnr 09-082M). One serum pool from eight healthy volunteers, who signed the informed consent form in adherence to the Declaration of Helsinki, was prepared at the Division of Clinical Chemistry, Department of Laboratory Medicine, Karolinska Institutet at Huddinge University Hospital, Stockholm, Sweden. Human samples were employed for comparative purpose.

### Cell lines and culture

J774A.1 murine macrophages were purchased from LGC Standards (Wesel, Germany). Fu5AH rat hepatoma cells were a gift from Prof Franco Bernini (Department of Food and Drug, University of Parma, Italy). Cell lines were cultured in RPMI medium or high glucose DMEM, respectively, at 37°C in a 5% CO_2_ atmosphere. All cell media were supplemented with 10% FBS and gentamicin (50 μg/ml).

### Plasma lipoproteins

Lipoproteins were separated from 2.5 μl of individual plasma samples by size exclusion chromatography, using a Superose 6 PC 3.2/300 column (GE Healthcare Bio-Sciences AB, Uppsala, Sweden). Lipoproteins were eluted as a fraction appearing in the exclusion volume of the sepharose column that contained CMs (if present) together with VLDL (named CM/VLDL), then LDL, and last HDL. TG, TC, unesterified cholesterol (UC), and PL concentrations were calculated after integration of the individual chromatograms (12, 13), generated by the enzymatic-colorimetric reaction with the respective kits: cholesterol oxidase phenol 4-aminoantipyrine peroxidase, glycerol phosphate oxidase-p-aminophenazone (Roche Diagnostics, Mannheim, Germany), and free cholesterol E, PL C (FujiFilm Wako Diagnostics, Mountain View, CA). The amount of esterified cholesterol was calculated by subtracting the UC from the TC. When cholesterol esters (CEs) were expressed in milligrams per deciliter, the values were multiplied for 1.67 in order to account for the fatty acid moiety.

Lipoproteins were also separated by sequential density ultracentrifugation in deuterium oxide-sucrose solutions (14). The lipoprotein fractions were resuspended in a buffer volume equal to the original sample volume. This procedure is suitable to small plasma volumes and allows for rapid analysis of the separated lipoprotein classes in electrophoretic and PG binding experiments. This method avoids interference of high salt concentrations, as would be the case using classic potassium bromide ultracentrifugation (14). Native electrophoresis of lipoprotein fractions was conducted with semiautomated equipment, and lipoproteins were stained by Sudan black (Sebia, Paris, France), by loading 10 μl of CM/VLDL/remnants (d = 1.006–1.019 g/ml), LDL (d = 1.019–1.063 g/ml), or HDL (d = 1.063–1.210 g/ml) into Hydragel 7 LIPO + lipoprotein (a) wells (Sebia, Paris, France). SDS-denaturing polyacrylamide gel electrophoresis of isolated lipoproteins as previously described (14) was used with the following modifications: 10 μl of Spectra Multicolor Broad Range Protein Ladder, HiMark Prestained Protein Ladder, or Spectra Multicolor Low Range Protein Ladder (Thermo Fisher Scientific Europe BV, The Netherlands) or 10–20 μl sample were added to each well of NuPAGE 4–12% Bis-Tris, Nupage 3–8% Tris-Acetate, or Novex 10% Tricine Protein Gel (Thermo Fisher Scientific Europe BV, The Netherlands) and run for about 50 min at a constant 200 V. Protein staining was performed with SimplyBlue Safe Stain (Thermo Fisher Scientific Europe BV, The Netherlands) and 20% NaCl solution, according to the manufacturer's protocol. Gel pictures were taken by Li-Cor Odissey Fc Imager and Image Studio 3.1 (LI-COR Biosciences – GmbH, Bad Homburg vor der Höhe, Germany) at 700 nm and 10 min exposure.

### Ex vivo binding of LDL to arterial PGs

This analysis was performed as previously described (15). In brief, human aortic PGs were isolated from the intima media of human aortas, and glycosaminoglycans from the PGs were quantified as markers of PG amounts. The wells of polystyrene 96-well plates were coated with 100 μl of PGs (50 μg/ml in PBS) by incubation at 4°C overnight. Wells were blocked with 1% BSA in PBS for 1 h at 37°C. Wells without PGs served as controls. To measure lipoprotein binding to the immobilized PGs, 1 μl of heavy water/sucrose-isolated LDL was added to the wells in a buffer containing 140 mmol/l NaCl, 2 mmol/l MgCl_2_, 5 mmol/l CaCl_2_, and 10 mmol/l MES, pH 5.5, and incubated for 1 h at 37°C. The wells were then washed with 10 mmol/l MES, 50 mmol/l NaCl, pH 5.5, and the amount of bound cholesterol was determined using the Amplex Red Cholesterol Kit (Thermo Fisher Scientific Europe BV, The Netherlands). The results are expressed as amount (micromoles) of bound-LDL-C per millimoles LDL-C added to the PG-coated wells.

### Cholesterol efflux capacity

Plasma specimens were thawed no more than twice on ice, and apoB-depleted serum was prepared (16). Aliquots were stored at −80°C. Cholesterol efflux experiments were performed as previously described (13) to evaluate CEC of both whole and apoB-depleted serum. Briefly, cells were incubated for 24 h with medium containing 1% FBS, [1,2-^3^H(N)]-cholesterol, and Sandoz 58-035 (2 μCi/ml). Cells were then incubated for 18 h with medium plus 0.2% BSA and Sandoz 58-035 (2 μCi/ml), adding compounds when appropriate. After this incubation, a set of cells were harvested with NaOH (1 mol/l) and counted by liquid scintillation. These cells provided baseline (time 0) values for total [1,2-^3^H(N)]-cholesterol content. The remaining cells were incubated with 1% (v/v) serum or 1.4% apoB-depleted serum (v/v) added to MEM for 4 h. Cell media were filtered to remove floating cells, and radioactivity in the supernatant was determined by liquid scintillation counting. Cholesterol efflux was calculated as follows: (cpm in medium at 4 h/cpm at time 0) × 100. J774.A1 cells cultured under basal conditions were used to evaluate the aqueous diffusion (AD). The ABCA1-mediated cholesterol efflux was the difference between the cholesterol efflux measured in J774 cells incubated with 8-(4-chlorophenylthio)-cAMP (0.3 mmol/l) and the cholesterol efflux measured in J774 cells cultured under basal conditions. The cholesterol efflux via scavenger receptor class B type I (SR-BI) was the difference between the cholesterol effluxes measured in Fu5AH cells cultured under basal conditions and in Fu5AH cells incubated with block lipid transporter 1 (10 μmol/l).

### Plasma LCAT and cholesteryl ester transfer protein activity assay

Plasma LCAT activity was measured using an exogenous standardized substrate as previously described (17). Briefly, the substrate was a reconstituted HDL made of apoAI, palmitoyloleylphosphatidylcholine and cholesterol at a weight ratio of 1:2.17:0.11 (corresponding to a molar ratio of 1:80:8), prepared by the cholate dialysis technique. Plasma and reconstituted HDL were mixed at a 1:10 volume ratio and incubated for 1 h at 37°C. UC was measured before and after the incubation by a standard enzymatic assay in the absence of cholesterol esterase. Absorbance at 510 nm was measured with a Synergy H1 multimode reader (BioTek Instruments, Inc., Winooski, VT).

Plasma cholesteryl ester transfer protein (CETP) activity was measured in 1.5 μl of sample using CETP Activity Assay Kit (Merck KGaA, Darmstadt, Germany), according to the manufacturer's instructions. Fluorescence was measured kinetically by Infinite F500 microplate reader (Tecan Trading AG, Switzerland).

### Statistical analysis

Continuous variables are presented as median (10th–90th percentile). Absolute numbers or percentages are summarized as categorical variables. Statistical analysis was performed using Statistica software (TIBCO, CA). Differences between the bears in winter to summer were determined by Wilcoxon signed rank test. When comparing humans with bears, the Kruskal-Wallis ANOVA was used followed by a multiple comparison post hoc test. For correlations, statistics were calculated by Spearman's rank *R*. Graphs were prepared using GraphPad Prism (GraphPad Software Inc., CA), and figures were prepared by Adobe Illustrator (Adobe Systems Inc., CA).

## Results

### Lipoprotein profiles and lipoprotein compositions

**Fig. 1:**
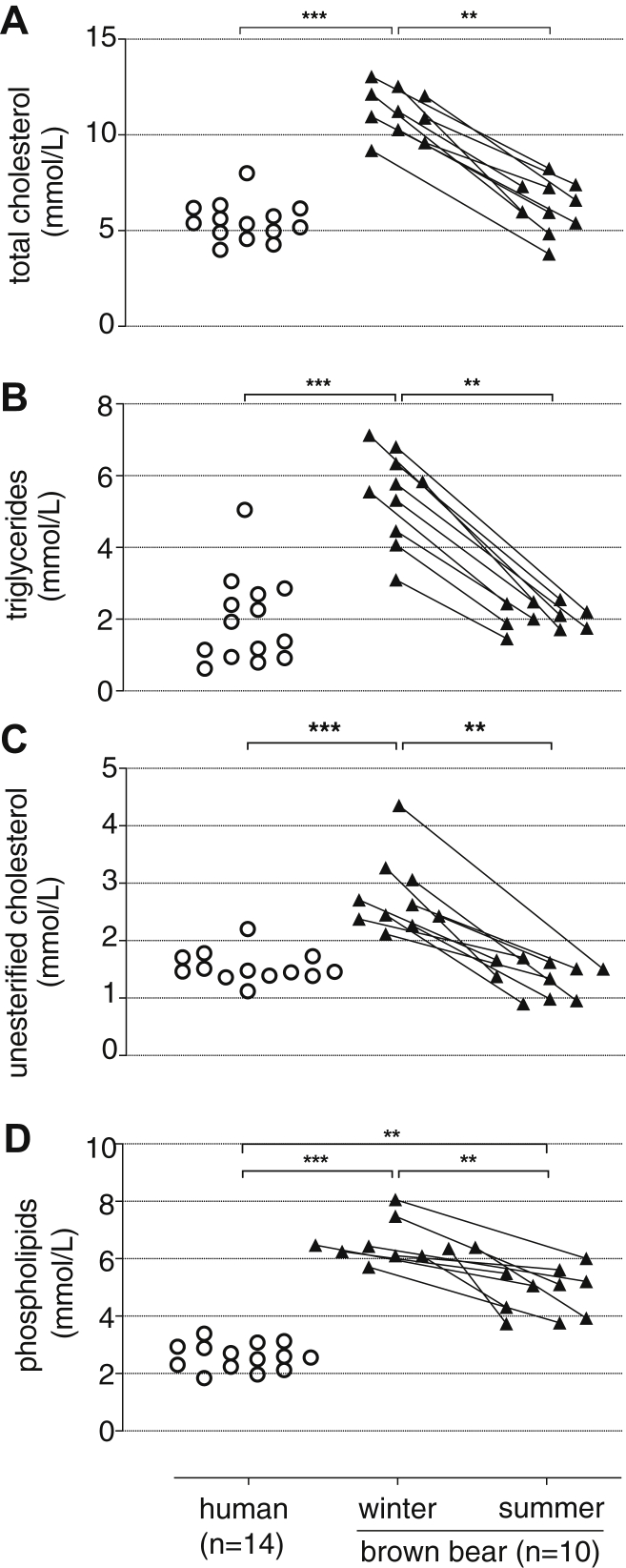
Plasma total lipid concentrations. Blood samples from n = 10 bears (black triangles) were taken during winter (February and March) and summer (June). N = 14 human serum samples (white circles) were run for comparative purposes. Plasma lipoproteins were separated by size exclusion chromatography (12, 13), and the TC (A) and UC (C), TGs (B), and PLs (D) concentrations were determined by a system allowing online detection. Data are plotted as individual values. Comparison between bears in winter versus summer was performed by Wilcoxon matched-pairs signed rank test, whereas comparison between human and bear in winter or bear in summer was done by Kruskal-Wallis ANOVA (at least *P* < 0.05) followed by multiple comparison test. Significances are indicated as follows: ∗∗*P* < 0.01, ∗∗∗*P* < 0.001.

**Fig. 2:**
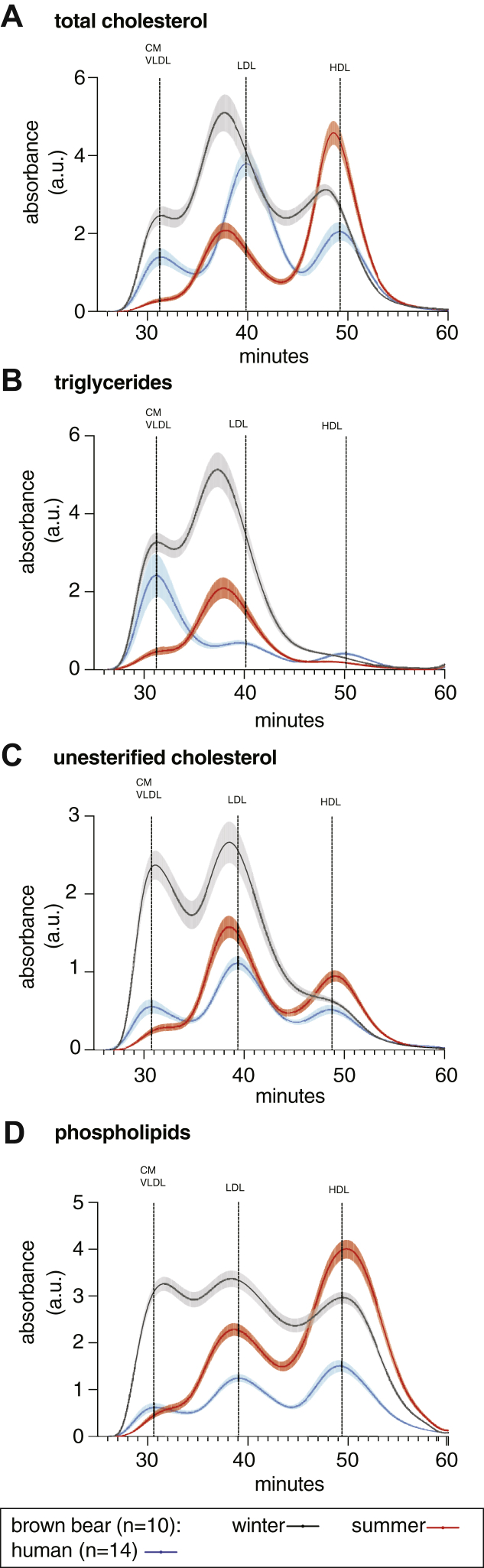
Plasma lipid lipoprotein chromatograms. Blood samples from n = 10 bears were taken during winter (February and March; black lines) and summer (June; red lines). N = 14 human serum samples (blue lines) were run for comparative purposes. Plasma lipoproteins were separated from 2.5 μl individual sample, by size exclusion chromatography (12, 13), and the individual chromatograms were generated by an enzymatic colorimetric reaction with the respective kits for TC (A) and UC (C), TGs (B), and PLs (E). Data are plotted as average chromatogram for each group (solid line) ± standard error of the mean (shadow around the solid line).

The plasma TC, TG, UC, and PL concentrations for bears in winter and summer and for human controls are presented in Fig. 1. In winter, when fasting, bears had more than 50% higher plasma levels of TC, UC, PL, and TG 30% higher, compared with summer. When compared with human healthy controls, all bear plasma lipid classes evaluated were higher only during hibernation (Fig. 1A–C), except for PL levels that were also higher in the summer (Fig. 1D). The lipid lipoprotein profiles by size exclusion chromatography (Fig. 2A) showed that TC in bears was carried mainly in the lipoprotein fraction corresponding to the human CM/VLDL and LDL in the winter (like in humans), whereas in the summer, the cholesterol was predominantly carried in HDL-sized particles. During the winter, TGs in bears were transported, unexpectedly, mostly in LDL (Fig. 2B). Figure 2C shows that the high content of UC in winter was distributed between CM/VLDL and LDL, but in the summer, the UC profile of bears was similar to that observed in humans. The elevated PL content of bears in winter (Fig. 2D) was similarly distributed in CM/VLDL, LDL, and HDL, whereas in the summer, the PLs were more prominent in LDL and HDL, similar to the distribution present in humans. As shown by their respective earlier elution times (Fig. 2), bear LDLs were larger than human LDLs.

Table 1 shows the concentration of CE, UC, TG, and PL in the different lipoprotein classes with the respective percentage of lipid composition. During hibernation, the major lipid classes in CM/VLDL increased more than 5–10 times above the summer levels in all 10 animals examined. Conversely, the increase of individual lipids in the LDL particles from hibernating bears was only one to two times higher, when compared with samples from the active summer period (Table 1). CE, TG, and PL levels of bear HDL did not differ between summer and winter. However, the bears in summer showed higher HDL-UC than in the winter (Table 1). In bears, the percentage of lipid composition of CM/VLDL, LDL, and HDL was not different during hibernation compared with the active states (Table 1). However, in winter, the bears had higher LDL-UC than in the summer. Major differences in both lipid concentration and percentage composition were observed between humans and bears (Table 1). Most noticeable were the higher levels of CE, TG, UC, and PL in the bear LDL during winter compared with humans. Also, bear LDLs were proportionally richer in TG and poorer in CE.

**Table 1:** Lipid class composition of plasma lipoproteins

Lipids	Lipoprotein								
	CM/VLDL/Remnants			LDL			HDL		
	Bear		Human (n = 14)	Bear		Human (n = 14)	Bear		Human (n = 14)
	Winter (n = 10)	Summer (n = 10)		Winter (n = 10)	Summer (n = 10)		Winter (n = 10)	Summer (n = 10)	
CE mmol/L	0.85 (0.39–1.28)∗∗	0.05 (0.02–0.25)^a^	0.67 (0.31–1.16)	4.64 (2.28–5.92)∗∗^,^^b^	1.40 (1.05–2.41)	2.15 (1.54–2.96)	2.84 (1.80–5.00)^a^	3.31 (2.20–4.47)^a^	1.08 (0.67–1.89)
CE mg/dL	55.18 (25.38–82.82)∗∗	3.13 (1.36–16.7)^a^	43.46 (20.15–74.72)	299.81 (147.14–382.40)∗∗^,^^b^	90.25 (67.94–155.67)	130.09 (99.19–178.88)	183.44 (115.95–321.41)^a^	213.43 (140.03–288.76)^a^	68.61 (43.53–112.04)
% weight	17 (7–20)^b^	11 (5–36)^c^	25 (13–36)	33 (23–41)^c^	23 (18–34)^a^	48 (46–52)	48 (39–59)^a^	44 (40–50)^b^	36 (31–41)
TG mmol/L	1.63 (1.11–2.12)∗∗	0.16 (0.05–0.36)^c^	0.95 (0.31–2.36)	3.71 (2.15–4.93)∗∗^,^^a^	1.65 (1.32–2.14)^c^	0.39 (0.29–0.59)	0.15 (0.12–0.18)^a^	0.12 (0.12–0.23)^c^	0.22 (0.19–0.34)
TG mg/dL	144.63 (97.87–187.7)∗∗	14.30 (4.69–32.16)^c^	84.14 (27.45–209.02)	328.55 (190.69–437.09)∗∗^,^^a^	146.62 (117.13–189.10)^c^	34.54 (25.69–52.26)	13.33 (10.45–16.34)^a^	13.99 (10.76–20.15)^c^	19.92 (16.82–30.11)
% weight	40 (36–45)	42 (20–73)	47 (29–63)	38 (32–41)^a^	37 (28–47)^a^	12 (10–20)	4 (2–5)^a^	3 (2–6)^a^	10 (7–18)
UC mmol/L	0.94 (0.61–1.30)∗∗^,^^b^	0.06 (0.02–0.12)^c^	0.37 (0.15–0.66)	1.48 (1.05–2.37)∗∗^,^^a^	0.79 (0.53–1.11)	0.77 (0.60–0.95)	0.26 (0.19–0.40)∗	0.50 (0.32–0.66)	0.33 (0.21–0.57)
UC mg/dL	36.43 (23.76–50.31)∗∗^,^^b^	1.89 (0.85–4.53)^c^	14.25 (5.61–25.41)	57.15 (40.70–91.74)∗∗^,^^a^	30.30 (20.50–42.89)	29.64 (23.32–36.62)	10.03 (7.44–15.45)∗	19.06 (12.45–25.64)	12.94 (7.93–22.20)
% weight	9 (8–13)∗^,^^c^	8 (3–11)	8 (6–9)	7 (6–10)^a^	7 (6–9)^c^	11 (10–12)	3 (2–3)∗∗^,^^a^	4 (3–4)^b^	7 (6–8)
PL mmol/L	1.65 (1.17–1.96)∗∗^,^^c^	0.14 (0.05–0.21)^b^	0.45 (0.22–0.63)	2.57 (1.85–3.43)∗∗^,^^a^	1.58 (1.17–2.01)^b^	0.97 (0.66–1.19)	2.19 (1.90–3.28)^c^	2.95 (2.42–3.97)^a^	1.15 (0.71–1.67)
PL mg/mL	127.5 (90.62–151.82)∗∗^,^^c^	10.9 (4.17–16.35)^b^	34.76 (16.70–48.87)	198.98 (142.62–264.98)∗∗^,^^a^	122.10 (90.23–155.03)^b^	75.23 (51.34–92.24)	169.25 (146.32–253.65)^c^	227.82 (187.08–306.84)^a^	88.76 (55.13–129.43)
%	32 (28–39)^a^	28 (18–42)^b^	18 (13–24)	23 (20–31) ∗∗	29 (26–35)	29 (22–31)	46 (36–54)	50 (45–54)^b^	46 (40–49)

Blood samples from bears were taken during winter (hibernation, February, March) and summer (June, free-ranging). N = 14 human serum samples were run for comparative purposes. Plasma lipoproteins were separated by size-exclusion chromatography, and the total cholesterol (TC) and unesterified cholesterol (UC), triglyceride (TG), and phospholipid (PL) concentrations were determined by a system allowing on-line detection. Cholesterol ester (CE) concentration was calculated as the difference between TC and UC. When the CE concentration is expressed in mg/dL, the value has been multiplied for 1.67 to account for the fatty acid moiety. Data are presented as the median (10^th^–90^th^ percentile) and indicate the concentration (mmoL/L or mg/dL) of each lipid species or their % weight composition within the lipoprotein particles. Comparison between bears in winter and bears in summer was performed by Wilcoxon matched pairs signed-rank test; ∗*P* < 0.05; ∗∗*P* < 0.01. Comparison between human and bears in winter or bears in summer was done by Kruskal-Wallis ANOVA (at least *P* < 0.05) followed by a multiple comparison test.

a *P* < 0.001.

b *P* < 0.05.

c *P* < 0.01.

### Plasma LCAT and CETP activity assay

Plasma LCAT activity seemed to be lower in bears than in humans, with a tendency to increase in from winter to summer (Table 2). These results were paralleled by a decreased plasma and HDL UC/TC ratio (supplemental Fig. S1). Moreover, we could not detect any CETP activity in the plasma from brown bears both in the winter and summer (supplemental Fig. S2).

**Table 2:** Plasma LCAT activity

Bear (code)	LCAT Activity (nmol/ml/h)	
	Winter	Summer
W1015	Bdl	4.3
W1017	4.5	16.7
W0904	Bdl	18.2
W1004	4.5	4.5
W1105	Bdl	7.8
W1104	6.4	4.3
W1110	3.4	14.2
Human (n = 14)	21.56 (6.3–32.0)	

Bdl, below detection limit.

Blood samples from n = 7 bears were taken during winter (February and March) and summer (June) and plasma prepared by centrifugation. N = 14 human serum samples were run for comparative purposes. LCAT activity was detected as previously described (17). Data for human are given as median (range).

### Electrophoretic properties of winter and summer lipoproteins

**Fig. 3:**
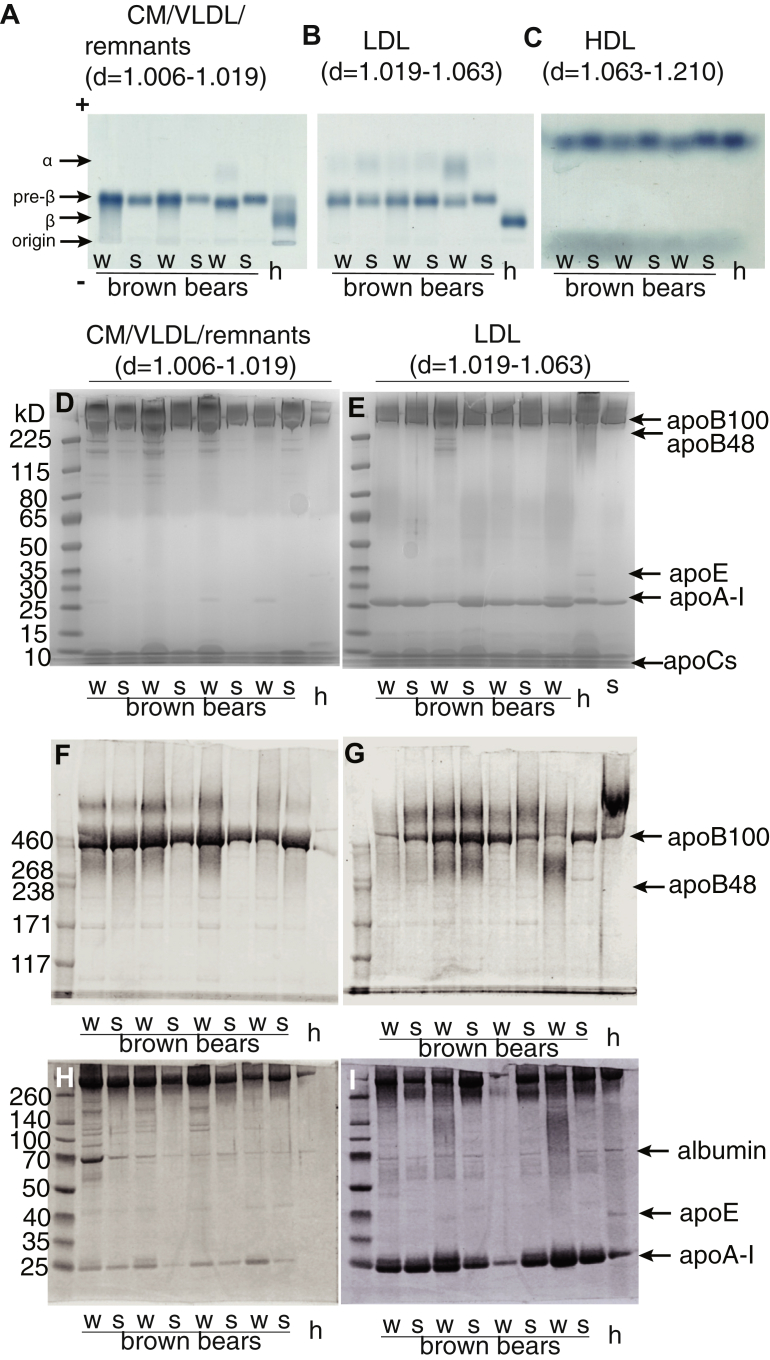
Native and denaturing gel electrophoresis of lipoproteins. Blood samples from n = 10 bears were taken during winter (February and March) and summer (June). N = 14 human serum samples or a human reference serum pool (n = 8) were run for comparative purposes. Plasma lipoproteins were separated by sequential density ultracentrifugation in deuterium oxide-sucrose solutions (14). For native gel electrophoresis, 10 μl of CM/VLDL/remnants (d = 1.006–1.019 g/ml; (A) LDL (d = 1.019–1.063 g/ml; (B) or HDL (d = 1.063–1.210 g/ml; (C) were loaded into an agarose gel and neutral lipids were stained by Sudan black. For denaturing gel electrophoresis: 20 μl of isolated CM/VLDL/remnants were loaded into a 4–12% Bis-Tris (D), 3–8% Tris-acetate (F), or 10% tricine gel well (H). About 20 μl of isolated LDL were loaded into a 4–12% Bis-Tris (E), 3–8% Tris-acetate (G), or 10% tricine (I) gel well. Proteins were stained by Coomassie blue. h, lipoprotein pool from apparently healthy humans (n = 8); S, bears sampled in the summer; W, bears sampled in the winter.

Native agarose electrophoresis of isolated bear lipoproteins revealed no major differences in electrophoretic mobility between winter and summer (Fig. 3). However, it should be noted that CM/VLDL/remnants (d = 1.006–1.019 g/ml) and LDL (d = 1.019–1.063 g/ml) share similar electrophoretic prebeta mobility (Fig. 3A, B), and both classes were much more anodic than human LDL (prebeta vs. beta). On the other hand, human and bear HDL (d = 1.063–1.21 g/ml) showed similar electrophoretic mobility (Fig. 3C). Thus, it appears that the brown bears had little or no beta lipoproteins. These electrophoretic properties were observed in all 10 bears. We also used SDS-polyacrylamide gel gradient electrophoresis to examine the most abundant apolipoproteins in the isolated lipoproteins. The CM/VLDL/remnants (Fig. 3D, F, H) seem to be particles containing apoB100, apoAI, and apolipoprotein E (apoE) as the most abundant apolipoproteins. This was also observed for the LDL fractions (Fig. 3E, G, I). Interestingly, and differently from human lipoproteins, we found that the bear apoB100-containing particles seem to be more enriched in apoAI than apoE (Fig. 3H, I).

### LDL binding to human arterial PGs

**Fig. 4:**
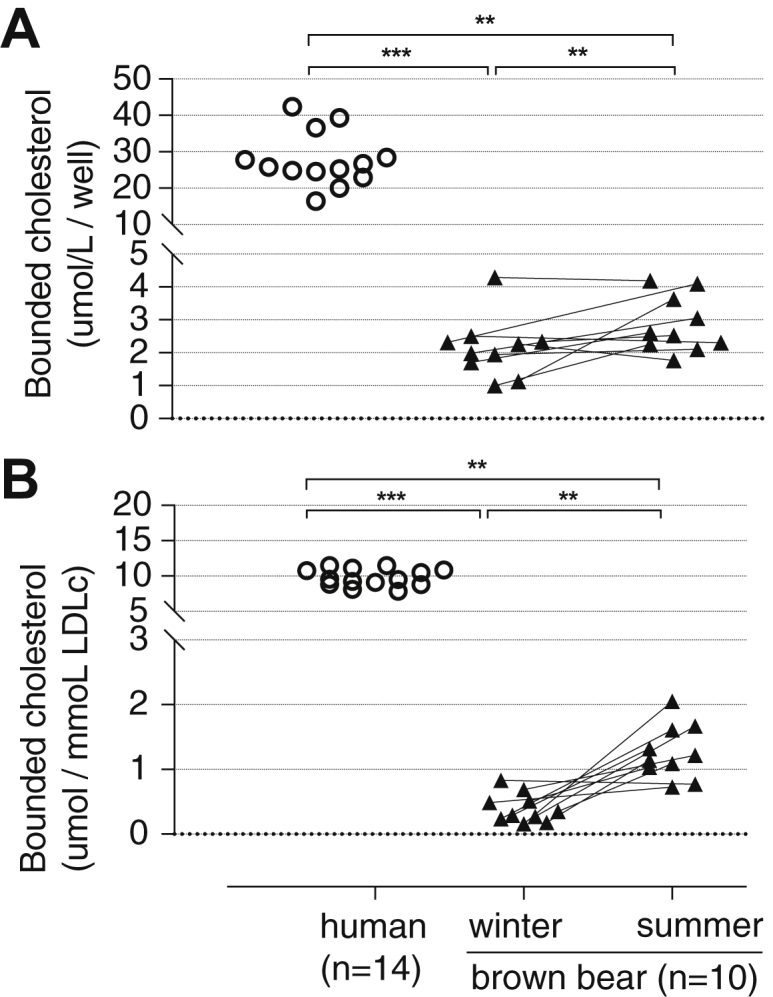
Binding of isolated LDL from brown bears to human arterial PGs. Blood samples from n = 10 bears (black triangles) were taken during winter (February and March) and summer (June). N = 14 human serum samples (white circles) were run for comparative purposes. Plasma lipoproteins were separated by sequential density ultracentrifugation in deuterium oxide-sucrose solutions (14). About 1 μl of isolated LDL (d = 1.019–1.063 g/ml) was tested to measure lipoprotein capacity to bind to human arterial PGs, isolated from human aortas, and plated in a 96-well plate. Results are expressed as micromoles of bound TC to the well (A) or as micromole of bound TC to the well divided by millimoles of LDL-TC added to the well (B). Data are plotted as individual values. Comparison between bears in winter versus summer was performed by Wilcoxon matched-pairs signed rank test, whereas comparison between humans and bears in winter or bears in summer was done by Kruskal-Wallis ANOVA (at least *P* < 0.05) followed by a multiple comparison test. Significances are indicated as follows: ∗∗*P* < 0.01, ∗∗∗*P* < 0.001.

The binding of isolated LDL to human arterial PGs was measured ex vivo. Although CE and UC content in bear LDL is higher in winter than in summer (Table 1), Fig. 4A shows that the winter LDL binds significantly less to human arterial PGs. These differences were observed even when adjusting the values of binding of LDL to PGs for the amount of LDL-TC added to the wells (Fig. 4B). Interestingly, both the winter and summer LDL from the bears bound 5 to 10 times less to the arterial PGs than the human LDL tested in the same analytical runs (Fig. 4).

### Plasma and HDL CEC

**Fig. 5:**
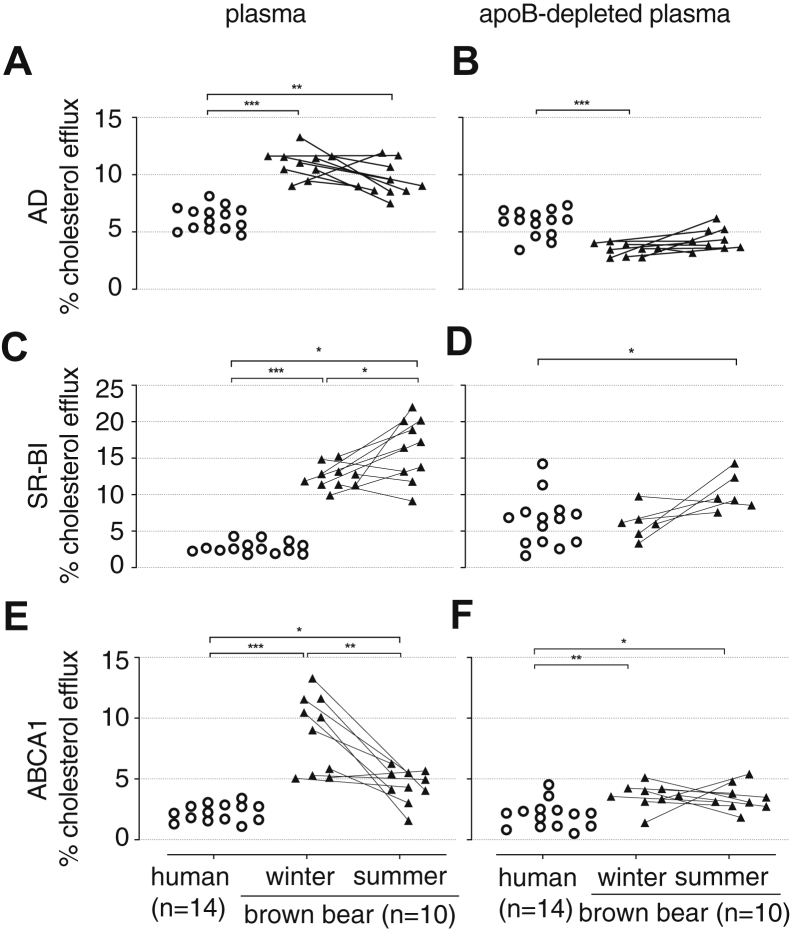
Plasma and HDL CEC. Cells were radiolabeled with [3H]-cholesterol for 24 h, equilibrated in a BSA-containing medium for 18 h, and exposed for 4 h to 1% (v/v) whole plasma (left panels) or 1.4% (v/v) apoB-depleted plasma (right panels). AD was assessed in J774A.1 cells, cultured under basal conditions (A, B); SR-BI-mediated efflux (C, D) was the difference between cholesterol efflux measured in Fu5AH cells cultured under basal conditions and Fu5AH cells incubated with block lipid transporter 1 (BLT-1). ABCA1-mediated cholesterol efflux (E, F) was the difference between the cholesterol efflux measured in J774A.1 cells, incubated with 8-(4-chlorophenylthio)-cAMP, and the cholesterol efflux measured in J774A.1 cells cultured under basal conditions. Blood samples from n = 10 bears (black triangles) were taken during winter (February and March) and summer (June). N = 14 human serum samples (white circles) were run for comparative purposes. Data are plotted as individual values. Comparison between bears in winter versus summer was performed by Wilcoxon matched-pairs signed rank test, whereas comparison between human and bears in winter or bears in summer was done by Kruskal-Wallis ANOVA (at least *P* < 0.05) followed by a multiple comparison test. Significances are indicated as follows: ∗*P* < 0.05, ∗∗*P* < 0.01, and ∗∗∗*P* < 0.001.

CEC was measured in whole plasma and apoB-depleted plasma, and the latter was lower in all the cholesterol efflux pathways studied (Fig. 5). Plasma CEC by AD (Fig. 5A) was almost double in bears compared with humans, but it did not differ between the summer and winter bear samples. The apoB-depleted plasma CEC (Fig. 5B) was higher in humans than in bears, but there was no difference in the CEC in the apoB-depleted plasma of hibernating and active bears. Plasma CEC via the SR-BI pathway was twice as high in bears compared with humans (Fig. 5C). Plasma CEC by SR-BI pathway was higher in summer than in winter, but this difference was lost when we evaluated the CEC using apoB-depleted serum (Fig. 5D). Human apoB-depleted plasma seems to have lower capacity to accept cholesterol via SR-BI than the bear apoB-depleted plasma specimen in summer (Fig. 5D). We also studied CEC via ABCA1, which was lower in serum from humans compared with bears in both summer and winter (Fig. 5E). Plasma CEC via ABCA1 from bears in summer decreased compared with the CEC in winter (Fig. 5E). When looking at apoB-depleted plasma CEC by this transporter, there were no differences between bears in summer and winter, but bears showed higher CEC than humans (Fig. 5F).

## Discussion

We believe that our results provide for the first time a mechanistic explanation that can contribute to the observed resistance to early atherogenesis of free-ranging brown bears, despite their high circulating levels of cholesterol and TG, especially during hibernation (5, 6). We found that LDL and HDL circulating in brown bears have functional and structural properties that could reduce their atherogenicity and protect them from vascular lipid infiltration and injuries. Bear LDL in the winter contained higher levels of TC and TG than in summer and when compared with humans (Fig. 1; Table 1). However, brown bear LDL showed a lower percentage content of CE than human LDL. Also, LDLs circulating in brown bears were approximately 2-fold richer in TG than the human LDLs. Thus, bear LDL, in both winter and summer, may deliver per particle less cholesterol to the arterial wall than human LDL. Moreover, bear LDL in both winter and summer were larger than human LDL (Fig. 2). This could be a consequence of their enrichment in TG (about 40% of total lipid cargo). In humans, the TG content carried by both small and large LDL separated by ultracentrifugation is only about 4–7% of the total lipid composition (18, 19). In our cohort of healthy humans, TG in LDL accounted for 2–5% of the total lipids. LCAT (20), an HDL-associated enzyme, catalyses the transfer of a fatty acid from PLs to UC resulting in the formation of CEs, which then move from the surface of the HDL particle to the core. The newly formed mature HDL can become the substrate for the CETP, which in turn affects TG-rich lipoprotein metabolism. Thus, we quantified brown bear plasma LCAT activity and found increased activity in summer compared with winter (Table 2). Those results were supported by a decrease in summer of the ratio between plasma and HDL UC/TC (supplemental Fig. S1), which is known, at least in humans, to be associated with plasma LCAT activity (21). Moreover, plasma LCAT seems to be lower in brown bears than in humans (Table 2). By exchanging TG in apoB-containing lipoproteins for CE in HDL, CETP is supposed to increase the cholesterol content of VLDL and LDL and thus reduce their TG cargo (22). In the present study, we could not detect any CETP activity in the plasma from bears in the winter or in the summer (supplemental Fig. S2). Taken together, these results provide one possible explanation for the TG enrichment of apoB-containing particles in these bears. Interestingly, in humans, large LDL particles are less associated with atherogenesis than small LDL, and several properties of LDL particles can explain this reduced atherogenicity (18, 19, 23, 24). Small LDL particles have a higher affinity for arterial intima PGs and can be more efficiently retained in the subendothelial space (7, 8, 18, 23). In addition, they appear to be more sensitive than large LDL to oxidative modifications and are more efficiently taken up by cultured human macrophages (7, 8, 23).

Retention of apoB100-containing lipoproteins, mainly LDL, within the arterial intima PGs appears to be the initial step leading to cholesterol accumulation, which triggers the inflammatory cascade and causes atherosclerosis (7, 8). The binding of human apoB100 lipoproteins to arterial PGs is mediated by specific sequences rich in arginine and lysine that have been identified ex vivo and animal models (25, 26). The amino acid sequence of brown bear apoB100 is more than 76% homologous with the human sequence over the full-length protein (Uniprot accession: P04114 for *Homo sapiens* and A0A3Q7Y8U4 for *Ursus arctos*; NCBI RefSeq accession: NP_000375.3 for *Homo sapiens* and XP_026375362.1 for *Ursus arctos*, see supplemental Tables S1 and S2 and supplemental Fig. S3). In humans, the apoB100 amino sequence containing the main arterial PG binding regions (amino acids 3,121–3,180 and 3,361–3,420) are polar segments with an excess of six positively charged lysine and arginine residues (7, 8, 25, 27, 28, 29, 30). These segments, located toward the C-terminal region of apoB100, are hydrophilic and surface exposed in human LDL (31), and this is also probable in the bear apoB100-containing lipoproteins. However, the same segments in *Ursus arctus* have only one excess positive charge (supplemental Fig. S4). Thus, apoB100-containing lipoproteins in bears should have a lower affinity for arterial PGs than the human LDL. This is supported by our results from the ex vivo assay of LDL-PG binding (Fig. 4). Importantly, it should be noted that the LDL of patients with clinical atherosclerosis (e.g., dyslipidemic patients with insulin resistance) ex vivo show a higher binding to arterial PGs than healthy controls (7, 8, 27, 32). For the first time, we showed that LDL in brown bears during winter displayed a lower binding than LDL in the summer, despite their higher CE and UC contents. More importantly, we demonstrated that LDLs from brown bears have much lower PG binding capacity than humans, regardless of the hibernating or active state. Liu *et al.* (28) in a population study concluded that polar bears (*Ursus maritimus*) diverged as a different species from brown bears only 400,000 years ago likely because of the extreme environmental pressures. Polar bears also have very high plasma levels of apoB-containing lipoproteins that appear to be a response to their very high fat diet all year around. In view of our findings, it would be interesting to compare the structural properties of polar bears VLDL and LDL and explore if they also have potentially atheroprotective properties as in brown bears.

In patients affected by cardiovascular diseases and in healthy controls, the LDL-C/apoB ratio, the apoB/TG ratio, and the LDL isoelectric point (i.e., surface charge) can predict the ex vivo complex formation of LDL with arterial PGs (LDL-PG affinity) with 70% accuracy (8, 33). Thus, the lower proportion of CE and UC, but higher proportion of TG, observed in brown bear LDL can explain their lower PG binding when compared with human LDL. Moreover, it is shown that human LDL that forms complexes with the highly negative sulfated glycosaminoglycans of the arterial PGs requires a beta electrophoretic mobility and a higher isoelectric point (pI = 5.54). It should be noted that in the binding assay used, the human VLDL with prebeta mobility (pI = 5.1) bind very little to arterial PGs at physiological pH (7, 8, 34). In the present study, we found that brown bear LDL had a prebeta and not a beta electrophoretic mobility (Fig. 3). Human LDL has a beta electrophoretic mobility similar to most nonhuman models of atherosclerosis (Fig. 3 and (35)). Indeed, lipoproteins that show agarose electrophoresis beta mobility, such as human LDL, have a negative surface charge from −4 to −7 mV, whereas those with prebeta mobility have a charge from −7 to −10 mV, such as the LDL of brown bears and human VLDL (35). The higher negative (or less positive) surface charge of the bear LDL particles might be caused by the mentioned differences in the surface-exposed apoB100 segments. Another possible reason for the prebeta mobility of the brown bear LDL could be the presence of nonesterified fatty acids bound to the bear LDL particle surface or dissimilarities in the content of associated exchangeable apolipoproteins (e.g., apoCIII, apoAI, and apoE) (35). From the analysis performed in this study, we are not able to provide any information regarding the different apoC isoforms bound to the bear lipoproteins. Nevertheless, we could show (Fig. 3) that the apoB100-containing particles (1.006 < d < 1.063), which both in winter and summer contained high levels of TG, seem to be more enriched in apoAI rather than ApoE. This apolipoprotein composition was more evident in the lipoprotein LDL particles with a density range between 1.019 and 1.063. It is a different condition than the human one, where apoAI is not associated to apoB100 but only to apoB48 when the CMs are in the lymph. Being such TG-rich apoB100-containing particles and having apoAI instead of apoE, this may lend to them being recognized to a lesser extent by scavenger receptors. Moreover, the presence of apoAI seems to reduce the ability to bind to PGs. Thus, from a lipoprotein point of view, brown bears seem to resemble dysbetalipoproteinemia (type III), but the absence of apoE and apoB48 makes their LDL size/density lipoproteins to become not atherogenic (the latter prevents a condition similar to an ApoE knockout).

The higher proportion of apoAI in bear LDL (d = 1.019–1.063) might also result from the presence of large HDL-1 in these density fractions. Indeed, the native agarose gel electrophoresis showed the presence of α-migrating lipoproteins (Fig. 3B). Moreover, these lipoprotein density fractions were characterized by an extra lipoprotein peak, with a size in between that of LDL and HDL, which was present in bears both in winter and summer and seems mainly composed of cholesterol and PLs (supplemental Fig. S5). This extra lipoprotein peak has been also described in other animal species as a large apoE-rich HDL (13, 36, 37). Nevertheless, in bears, the amount of apoE seems to be relatively very small, whereas the apoAI is predominant.

Cellular cholesterol efflux to HDL is one of the initial steps in reverse cholesterol transport, and it occurs by several mechanisms. These rely upon different HDL subclasses that are specific acceptors for individually identified mechanisms (38, 39): *a*) AD (which includes unknown transporters) to mature HDL; *b*) SR-BI-mediated efflux to mature HDL; *c*) ABCG1-mediated efflux to mature HDL and preβ-HDL; and *d*) ABCA1-mediated efflux to apoAI, preβ-HDL, and small HDL particles (16, 40, 41, 42). To our knowledge, this is the first time that CEC as metric of HDL function has been measured in brown bears and compared with that in humans with the same methods. The cell models used in the efflux assay are heterologous and may interact differently with plasma lipoprotein than with bear extrahepatic cells. Nevertheless, all the serum specimens were tested in the same culture conditions and well-established cell models employed for measuring CEC both in human and preclinical studies (13, 16, 43). Hence, we could differentiate the major mechanisms driving cell cholesterol efflux and test both plasma and apoB-depleted plasma in order to determine differences related to species and seasons. The use of apoB-depleted plasma or isolated HDL as cholesterol acceptor is an established method for the measurement of HDL CEC. Indeed, the interferences of apoB-containing particles in the efflux process are avoided. Nevertheless, as also suggested by others (40), apoB-depleted plasma does not resemble the in vivo situation since both apoB- and apoAI-containing lipoproteins are always present in plasma. It is also known that plasma albumin can act as cholesterol acceptor from cells (41). We could show, indeed, that plasma CEC was higher than apoB-depleted plasma CEC in all the different efflux pathways and bears had a higher plasma CEC compared with humans. Interestingly, when testing HDL CEC, by depletion of the plasma specimen of the apoB-containing particles, bears, both in summer and winter, showed higher CEC via ABCA1, the pathway that determines the capacity of plasma specimens with similar HDL-C to remove cholesterol from macrophages (44).

Our study should be considered with the following caveats. The free-ranging brown bears studied were subadult animals, whereas the absence of arterial atherosclerosis was previously assessed in adult bears (6). On average, primiparity occurs at the age of 4.3 years in brown bears and senescence at age 23. On this scale, a 12-year-old brown bear equates to a 45–55-year-old human. Given the absence of even the earliest signs of atherosclerosis (fatty streaks) in adult bear (6), we find it reasonable to infer that it is likely that brown bears have structural properties of lipoproteins with low atherogenic profile even when they circulate with high levels of cholesterol and TGs in apoB100-containing particles. Moreover, the bears in the current study were 2–3 years old at time of sampling: thus, circulating high lipids are already present early in the life, and most likely remain high in adulthood. All bears were from the same geographical area. Moreover, our study focused only on lipoprotein composition and selected lipoprotein function: other pathways and mechanisms that may also differ in brown bears and humans (e.g., phenotype of circulating monocytes, circulating micro-RNA, composition of arterial PGs, protective inflammatory responses, hemodynamic differences) might contribute in reducing atherogenesis in brown bears.

In conclusion, despite high TC and TG levels in apoB-containing lipoprotein, the brown bear lipoprotein profile appears to be less atherogenic than that of humans, thus resulting in a vasculoprotective effect. This could be associated with the low LDL affinity for PGs, secondary to their increased TG and PL, and to their low positive surface charge. In addition, the higher plasma CEC may further reduce the atherogenicity of the bear lipoprotein profile, by controlling cholesterol accumulation in the arterial intima and thus preventing the early stages of atherosclerosis development. These atheroprotective and vasculoprotective mechanisms of the bear lipoprotein profile seem to be driven by composition- and structure-modulated functions.

## Data availability

All data generated or analyzed during this study are included in this published article (and its supplemental data files). The data sets generated during and/or analyzed during the current study are available from the corresponding authors on reasonable request.

## Supplemental data

This article contains supplemental data (12, 13, 14, 28, 30).

## Conflict of interest

From March 2014 to March 2017, AstraZeneca employed M. P. as a senior postdoc. M. P. is a founder and co-owner of Lipoprotein Research Stockholm AB, together with P. P., who is the CEO of this company. AstraZeneca employs E. H.-C. Part of the lab costs has been financed by AstraZeneca in the form of unrestricted financial support. No funding agency had any role in the design and conduct of the study, in the collection, management, analysis, or interpretation of the data, or in the preparation, review, or approval of this work. All other authors declare that they have no conflicts of interest with the contents of this article.
