# Supplementary material for: Vasculoprotective properties of plasma lipoproteins from brown bears (Ursus arctos)
Source: J Lipid Res. 2021 Mar 11;62:100065. doi: 10.1016/j.jlr.2021.100065 (PMC8131316; doi:10.1016/j.jlr.2021.100065)
Supplement: Supplemental Table S1, S2 and Figures S1 to S5 [file mmc1.pdf]

SUPPLEMENTAL INFORMATION:

Vasculoprotective Properties of Plasma Lipoproteins from Brown Bears (*Ursus arctos)*

Matteo Pedrelli[1,2,*], Paolo Parini[1,3,4], Jonas Kindberg[5,6], Jon M. Arnemo[6,7], Ingemar Bjorkhem[1], Ulrika Aasa[8], Maria Westerstahl[9]; Anna Walentinsson[2], Chiara Pavanello[10], Marta Turri[10], Laura Calabresi[10], Katariina Öörni[11], Gérman Camejo[1], Ole Fröbert[6,12], and Eva Hurt-Camejo[1,2,*]

1. Division of Clinical Chemistry, Department of Laboratory Medicine, Karolinska Institutet, Stockholm, Sweden
2. Translational Science & Experimental Medicine, Research and Early Development, Cardiovascular, Renal and Metabolism (CVRM), BioPharmaceuticals R&D, AstraZeneca, Gothenburg, Sweden
3. Metabolism Unit, Department of Medicine, Karolinska Institutet, Stockholm, Sweden
4. Theme Inflammation and Infection, Karolinska university Hospital, Stockholm, Sweden
5. Norwegian Institute for Nature Research, Trondheim, Norway
6. Swedish University of Agricultural Sciences, Department of Wildlife, Fish, and Environmental Studies, Umeå, Sweden
7. Department of Forestry and Wildlife Management, Inland Norway University of Applied Sciences, Campus Evenstad, Koppang, Norway
8. Department of Community Medicine and Rehabilitation, Umeå University, Umeå, Sweden
9. Division of Clinical Physiology, Department of Laboratory Medicine, Karolinska Institutet, Stockholm, Sweden
10. Centro Enrica Grossi Paoletti, Dipartimento di Scienze Farmacologiche e Biomolecolari, Università degli Studi di Milano, Milan, Italy
11. Atherosclerosis Research Laboratory, Wihuri Research Institute, Helsinki, Finland
12. Örebro University, Faculty of Health, Department of Cardiology, Örebro, Sweden

Uniprot Identifier	APOB_MOUSE	APOB_RAT	A0A452SJS0_URS	A0A3Q7Y8U4_UR	A0A384CVD9_UR	A0A287AG13_PIG	E1BNR0_BOVIN	APOB_HUMAN	Species
**APOB_MOUSE**	100	89	67	67	67	68	67	71	Mus musculus (mouse)
**APOB_RAT**	89	100	67	67	66	67	66	70	Rattus norvegicus (rat)
**A0A452SJS0_URSAM**	67	67	100	99	99	77	75	76	Ursus americanus (american black bear)
**A0A3Q7Y8U4_URSAR**	67	67	99	100	99	77	75	76	Ursus arctos (brown bear)
**A0A384CVD9_URSMA**	67	66	99	99	100	77	75	76	Ursus maritimus (polar bear)
**A0A287AG13_PIG**	68	67	77	77	77	100	80	76	Sus scrofa (pig)
**E1BNR0_BOVIN**	67	66	75	75	75	80	100	74	Bos taurus (bovine)
**APOB_HUMAN**	71	70	76	76	76	76	74	100	Homo sapiens (human)

Supplemental Table S1. Percentage amino acid sequence identity of apoB-100 between species

Type	Description	Position(s) incl. signal peptide	Position(s) excl. signal peptide	Length
Signal peptide	-	1 - 27	-	27
Chain	Apolipoprotein B-100	28 - 4563	1 - 4536	4536
Chain	Apolipoprotein B-48	28 - 2179	1 - 2152	2152
Domain	Vitellogenin	46 - 672	19 - 645	627
Region	Heparin-binding	32 – 126	5 - 99	95
Region	Heparin-binding	232 – 306	205 - 279	75
Region	Heparin-binding	902 – 959	875 - 932	58
Region	Heparin-binding	2043 – 2178	2016 - 2151	136
Region	Heparin-binding	3161 – 3236	3134 - 3209	76
Region	Basic (possible receptor binding region)	3174 – 3184	3147 - 3157	11

Region	LDL receptor binding	3373 – 3393	3346 - 3366	21
Region	Heparin	3383 – 3516	3356 - 3489	134
Region	Basic (possible receptor binding region)	3386 – 3394	3359 - 3367	9

Supplemental Table S2. Functional features in human apolipoprotein B-100 protein (UniProtKB - P04114 APOB_HUMAN)

Supplemental Figure S1

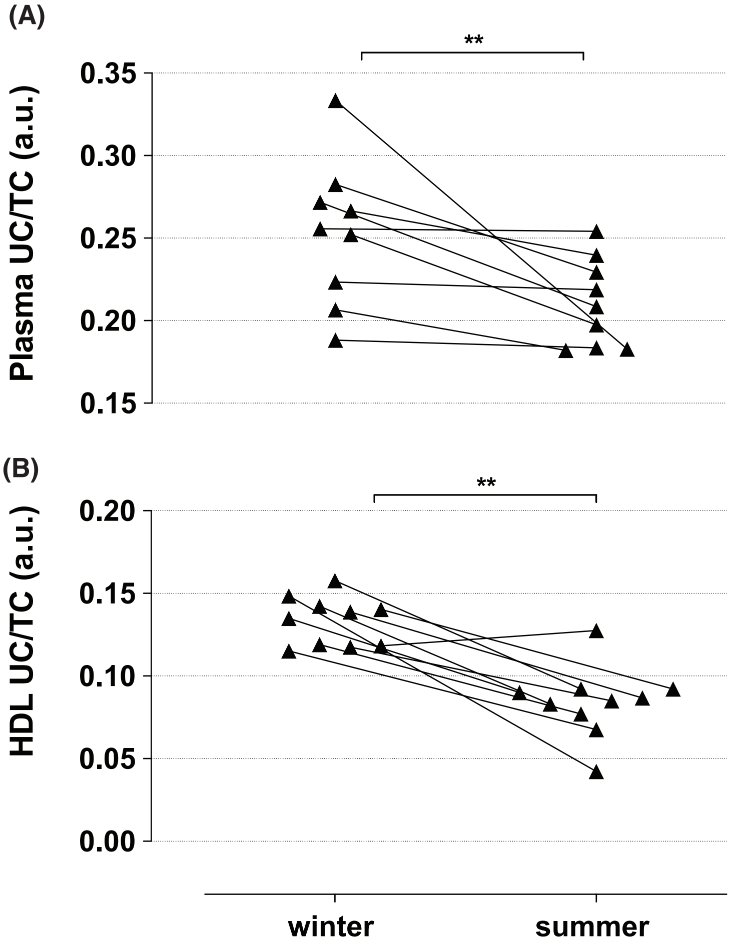

Supplemental Figure S1. Plasma ratio between unesterified and total cholesterol

Blood samples from n=10 bears were taken during winter (February, March) and summer (June) and plasma prepared by centrifugation. N=14 human serum sampls were run for comparative purposes. Plasma lipoproteins were separated by size exclusion chromatography (1), and the total (TC; A) and unesterified (UC; C) concentration was determined by a system allowing on-line detection. Data are plotted as individual values. Comparison between bears in winter vs summer was performed by Wilcoxon Matched Pairs test. Significances are indicated as follows: ** p<0.01, *** p<0.001.

Supplemental Figure S2

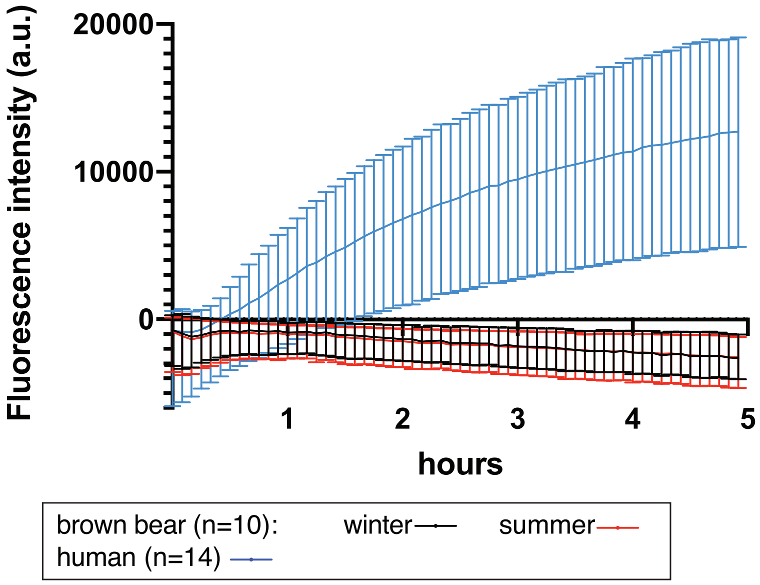

Supplemental Figure S2. Plasma CETP activity measurment

Blood samples from n=10 bears were taken during winter (February, March) and summer (June) and plasma prepared by centrifugation. N=14 human serum samples were run for comparative purposes. CETP activity was detected florimetrically every 5 min, in 1.5 uL of sample. The data are plotted for each reading point as median (range) in the bear (n=10) winter (black line), summer (red line) and human (n=14; blue line) groups.

Supplemental Figure S3

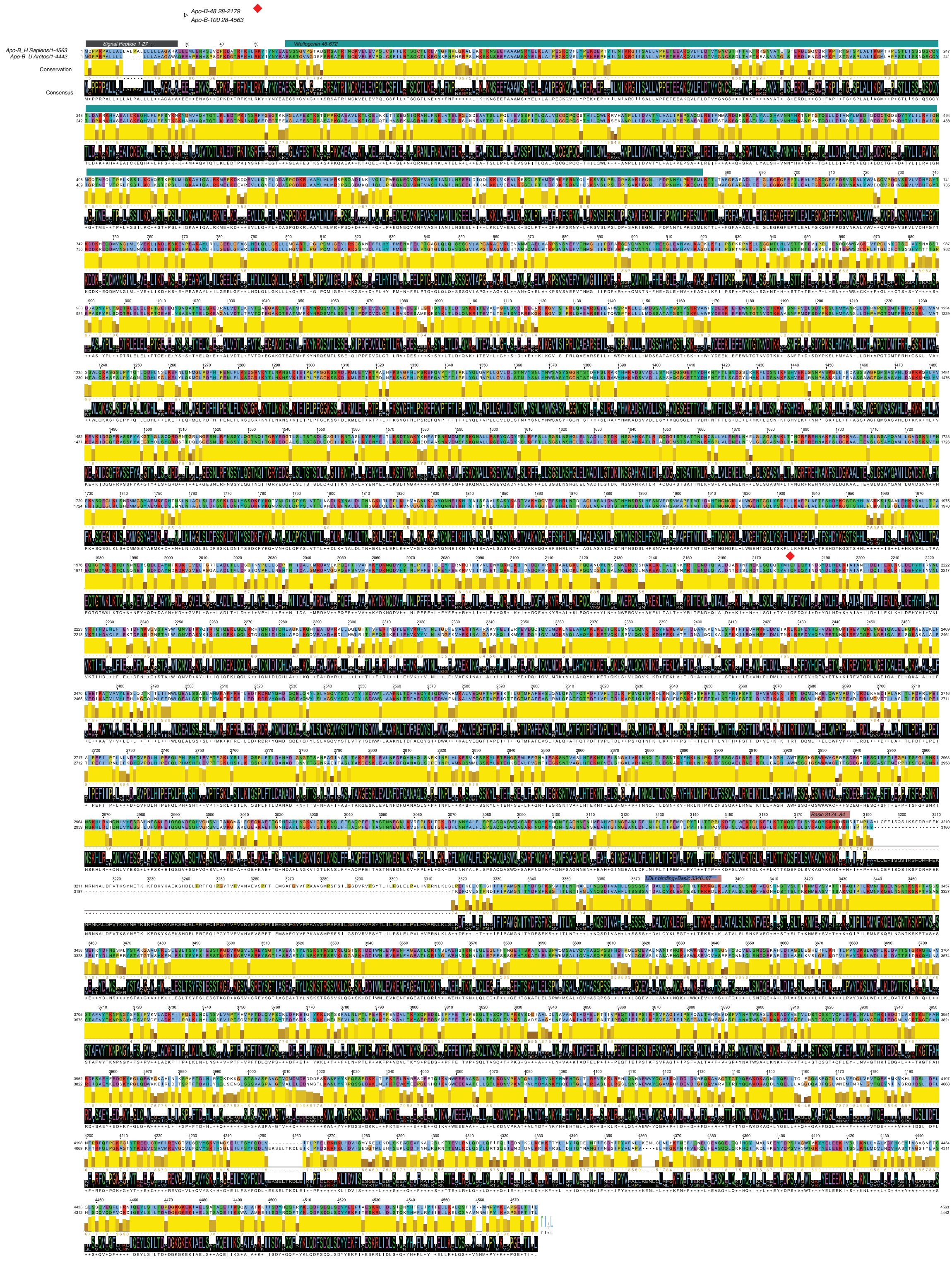

Supplemental Figure S3. Alignment of human and brown bear ApoB-100 proteins

Supplemental Figure S4

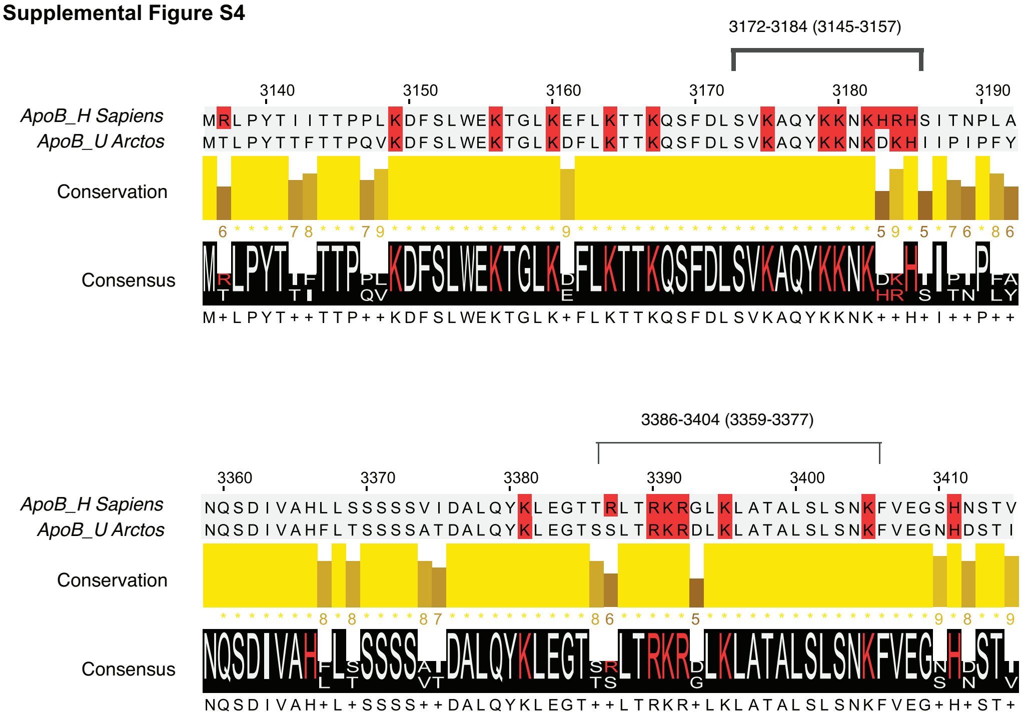

Supplemental Figure S4. Segment of apoB-100 responsible for the binding with proteoglycans in the arterial intima. The positional informations obtained from two studies (2) was combined and compared to the amino acid sequence of brown bear apoB-100, which is more than 76 % homologous with the human sequence over the full-length protein.

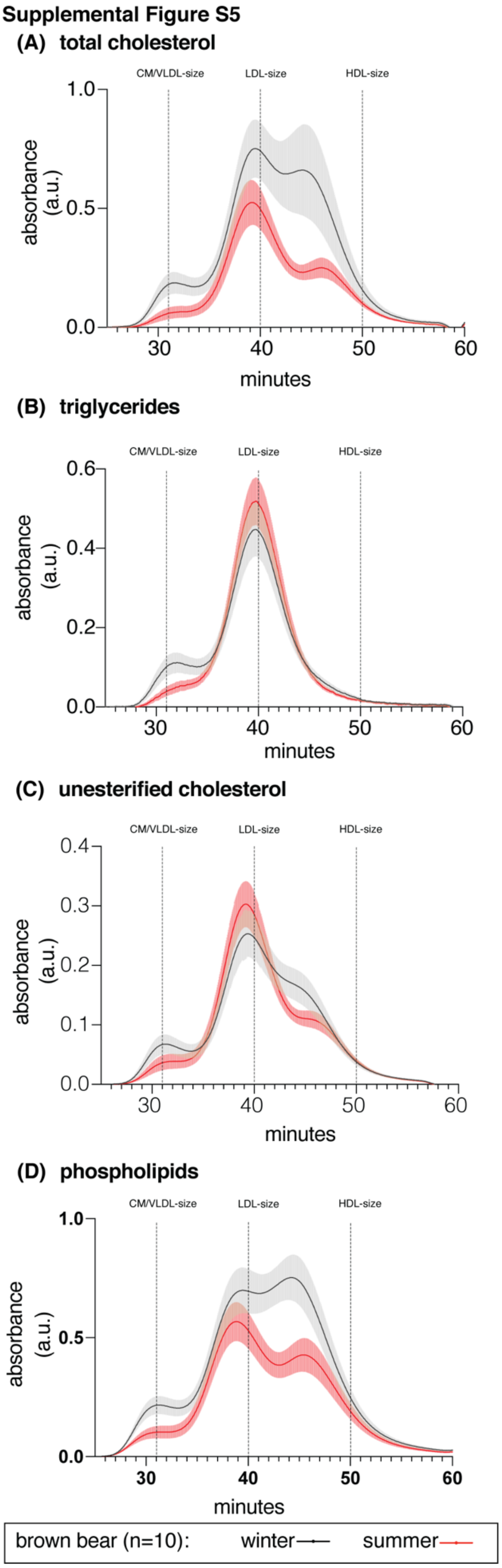

Supplemental Figure S5. Bear LDL (d=1.019-1.063) lipid profile

Blood samples from n= 10 bears were taken during winter (February, March; black lines) and summer (June; red lines). Plasma lipoproteins were separated by sequential density ultracentrifugation in deuterium oxide-sucrose solutions (3) and subsequently the LDL (d=1.019-1.063) were separated from each individual sample, by size exclusion chromatography (1). The chromatograms were generated by the enzymatic-colorimetric reactions with the respective following kits for total (TC; A) and unesterified (UC; C) cholesterol, triglycerides (TG; B), and phospholipids (PL; E). Data are plotted as average chromatogram for each group (solid line) ± standard error of the mean (shadow around the solid line).

Supplemental References

1. Olsson, U., G. Camejo, E. Hurt-Camejo, K. Elfsber, O. Wiklund, and G. Bondjers. 1997. Possible functional interactions of apolipoprotein B-100 segments that associate with cell proteoglycans and the ApoB/E receptor. *Arteriosclerosis, thrombosis, and vascular biology* **17**: 149-155.

2. Olsson, U., G. Camejo, S. O. Olofsson, and G. Bondjers. 1991. Molecular parameters that control the association of low density lipoprotein apo B-100 with chondroitin sulphate. *Biochim Biophys Acta* **1097**: 37-44.

3. Parini, P., L. Johansson, A. Broijersen, B. Angelin, and M. Rudling. 2006. Lipoprotein profiles in plasma and interstitial fluid analyzed with an automated gel-filtration system. *Eur J Clin Invest* **36**: 98-104.

4. Pedrelli, M., P. Davoodpour, C. Degirolamo, M. Gomaraschi, M. Graham, A. Ossoli, L. Larsson, L. Calabresi, J. A. Gustafsson, K. R. Steffensen, M. Eriksson, and P. Parini. 2014. Hepatic ACAT2 knock down increases ABCA1 and modifies HDL metabolism in mice. *PLoS One* **9**: e93552.

5. Stahlman, M., P. Davidsson, I. Kanmert, B. Rosengren, J. Boren, B. Fagerberg, and G. Camejo. 2008. Proteomics and lipids of lipoproteins isolated at low salt concentrations in D2O/sucrose or in KBr. *J Lipid Res* **49**: 481-490.
